# Modern Sociology

**Published:** 1901-09-21

**Authors:** 


### Modern Sociology.

### THE CASE OF THE SHOP ASSISTANT.

### III.—Fines and Deductions.

Though long hours of labour are at the bottom of the troubles of the assistant, there are other conditions which make the life an unhealthy one from whatever point of view it is considered. A Chelsea shopkeeper, in the course of a conversation we had with him, said that the modern tendency was to turn the assistants into machines. "And that," he added, "is surely retrogressive."

One of the means employed for the purpose of turning out the machine is the system of fines, and to glance down the list in force at many shops is to wonder how the assistant keeps his or her head at all. The exclamation uttered by Miss H. Jastrow, late secretary of the Berlin Women Clubs and Shop Assistants' Association, is not surprising. "What kind of men are they," she asks, "who work under such rules, and what work can be expected under such conditions? Fines such as are enforced in English houses do not exist (in German houses), nor would a firm employ people for whom such rules are necessary."

"There are 99 fines at our house," one girl tells us, "but they are not all enforced." Among those that are, however, are some that no other class of workers would tolerate, and that are, moreover, distinctly illegal. Half-a-crown is inflicted for speaking to the servants at meals on any other subject than the food (one could understand this better if the food itself were the tabooed topic); and the same for leaving the shop without first asking permission of the shop-walker or buyer. This is an instance of a whole class of grievances which more especially affect women. Where the buyer is a woman there is no hardship; but circumstances can readily be imagined in which—call it false modesty or not—a girl naturally shrinks from asking permission from a man. We learn from a lady doctor who has had great experience among shop-girls that it is not only carelessness on the girls' part that leads them to come down to work for the day in ignorance of their state of health; it is also that they suffer so commonly from anæmia that it is impossible to forecast events. "I have sometimes lain down behind the counter," one shop assistant told us; "the misery of standing was so great, and I could not ask if I might go." Fines for lateness in coming to work are common; mistakes in bills, sending goods to wrong addresses, omitting to replace paper, string, and pins in their places, giving wrong measure, presenting bad money at the desk, taking wrong change from the cashier, not wrapping up each small article in paper before packing it with other things in a parcel, leaving small wares in front of customers, moving a customer once seated, using the word "guaranteed" about goods which may afterwards disappoint the customer, omitting to dust every box (a fine for each omission), and, the greatest sin of all, allowing the customer to leave the shop without buying something—all these offences are actually fined in many shops. Some firms, not content with a fairly exhaustive list, add that anything done, or permitted to be done, contrary to the interests of the firm, not already specified, may be fined at the discretion of the firm, the amount to be left to the shop-walkers and buyers, a species of "omnibus" fine beautifully comprehensive.

What is the legal position of the shopkeeper with respect to fines? The Truck Act of 1896 applies, as it specially states, to "the case of a shop assistant in like manner as it applies to the case of a workman." Deductions or payments in respect of fines are not to be made unless the fine imposed "is in respect of some act or omission which causes or is likely to cause damage or loss to the employer, or interruption or hindrance to his business." The amount of the fine must be fair and reasonable, having regard to all the circumstances of the case; and particulars in writing, showing the acts or omissions in respect of which the fine is imposed, and the amount thereof, are to be supplied to the workmen on each occasion when a deduction or payment is made"; so that the "omnibus fine" above quoted is illegal. It seems clear that shopkeepers regard themselves as free to break the Truck Act with complete impunity, and this view has been encouraged by the statement of the late Home Secretary that he did not intend to enforce this Act in shops. "One of the most objectionable features in the system," says Miss Clara Collet, speaking of a case she investigated, "was that their fines were given to the clerk who inflicted them."

Deductions, though different from fines, are equally illegal under the Act of 1896, and are another fruitful source of revenue. At one shop we understand that a most unreasonable system prevails, viz., one farthing is deducted from every shilling paid to the assistant in salary or received in premiums. This is given to the cashier. Now a first hand in a department, *e.g.*, hosiery and gloves, can earn £30 in salary per year, and perhaps £25 in premiums. As first hand she has the first choice of customers; those below her in rank make less. Out of her earnings alone, therefore, £1 2s. 11d. a year is retained for the purpose of adding to the 5s. a week earned by the cash girls. This is specified on the sheet given to the assistant when engaged, and one girl questioned saw no injustice in it until it was pointed out to her that an exactly parallel case would be that of a lady engaging servants and telling them it was her custom to keep back part of their wages to pay the boot boy. It is not only in retail prices that the cumulative value of farthings is recognised in the distributive trade. Deductions for the house doctor are a serious grievance, and frequently the assistant pays the amount, usually a shilling a month, and chooses his or her own medical adviser. It has been pointed out that this is not a very dignified position for a doctor to hold, and that he is handicapped unpleasantly by the knowledge that a long sick list would not advance his interest with the firm employing him. The writer of "Life in the Shop" gives the monthly deductions of a shop in Chelsea where 250 assistants are employed as follows:—

| | s. | d. |
|---|---|---|
| Doctor ... ... ... ... ... | 1 | 0 |
| Boots ... ... ... ... ... | 1 | 0 |
| Library ... ... ... ... ... | 1 | 0 |
| Early Closing and Premium Sheets ... | 0 | 5 |
| | 3 | 5 |

There is a certain irony in deducting for a subscription to the Early Closing Association, when no guarantee is given that the assistant shall benefit by it. Premiums, fines, and deductions are checked over against each other in the cash office, and the net result is handed to the assistant at the end of the month. "Would you not prefer a fixed higher salary," we asked one girl, "instead of an uncertain amount at the end of the month?" "Yes," she answered hesitatingly, "of course it would cover slack seasons if it was good enough, but I don't think girls would take so much interest in their work without the premiums." By such artificial means is the machine turned out and rendered effective for the employers' interest.

At a public meeting of shop assistants recently the following questions were asked by Mr. J. Macpherson, general secretary to the Shop Assistants' Union:—"Why should shop assistants pay for blacking and not get their boots blacked? Why should they pay 1s. a month for breakages and not break anything? Why should they pay 1s. a month for a library, and then, when the firm wanted to turn itself into a limited liability company, find the library described as a 'valuable asset' to be taken over?" Enough has been said to show that in the least organised trade the assistants do not even reap the benefit of such scanty laws as have been passed for their benefit. A clause in Sir Charles Dilke's Bill provides that the Truck Act of 1896 shall be enforced by H.M. Inspectors of Factories and Workshops.

